# Avidity of Polyclonal Antibodies to Foot-and-Mouth Disease Virus in Bovine Serum Measured Using Bio-Layer Interferometry

**DOI:** 10.3390/v14040714

**Published:** 2022-03-29

**Authors:** Andrew E. Shaw, Alison Burman, Amin Asfor, Emiliana Brocchi, Santina Grazioli, Clare Browning, Anna Ludi, Tobias J. Tuthill, Donald P. King

**Affiliations:** 1 The Pirbright Institute, Pirbright, Woking GU24 0NF, UK; alison.burman@pirbright.ac.uk (A.B.); amin.asfor@pirbright.ac.uk (A.A.); clare.browning@pirbright.ac.uk (C.B.); anna.ludi@pirbright.ac.uk (A.L.); toby.tuthill@pirbright.ac.uk (T.J.T.); donald.king@pirbright.ac.uk (D.P.K.); 2 Department of Pathology and Infectious Diseases, Faculty of Health and Medical Sciences, School of Veterinary Medicine, University of Surrey, Guilford GU2 7AL, UK; 3 Istituto Zooprofilattico Sperimentale della Lombardia e Dell’Emilia Romagna “Bruno Ubertini”, 25124 Brescia, Italy; emiliana.brocchi@gmail.com (E.B.); santina.grazioli@izsler.it (S.G.)

**Keywords:** foot-and-mouth disease virus, antibodies, bio-layer interferometry, avidity, vaccines

## Abstract

Foot-and-mouth disease (FMD) is a disease of cloven-hoofed livestock caused by FMD virus (FMDV). FMD can be controlled through the use of inactivated vaccines, and it is well established that the protection afforded by FMD vaccines correlates strongly with neutralising antibody titres. However, the overall strength of binding, referred to as avidity, is also an important parameter with respect to the ability of antibodies to neutralise virus infection, and there is evidence that avidity can affect the level of protection afforded by FMDV vaccines. Here, as an alternative to modified enzyme-linked immunosorbent assays (avidity ELISAs) incorporating a chaotropic wash step, we used bio-layer interferometry (BLI) to measure the avidity of bovine polyclonal antibodies against FMDV capsids. We conducted preliminary experiments using recombinant FMDV capsids, as well as peptides representing antigenic loops, to demonstrate that the binding of monoclonal antibodies targeting specific antigenic sites could be detected using BLI. Subsequent experiments using polyclonal sera derived from FMD vaccinated cattle provided evidence of a positive correlation between the neutralising titre of the serum and the avidity as measured by BLI. Furthermore, we observed an increase in BLI avidity, as well as in the titre, in vaccinated animals upon challenge with the live virus.

## 1. Introduction

Foot-and-mouth disease (FMD) is caused by the picornavirus FMD virus (FMDV) and affects wild and domestic members of the Artiodactyla, including cattle, sheep, pigs and buffalo. FMDV infection causes a dramatic reduction in productivity and is globally estimated to be responsible for annual losses of USD 8.5–22.5 billion [1]. 

FMDV exists as seven immunologically distinct serotypes: O, A, Asia1, C, South African territories (SAT)1, SAT2 and SAT3, although recent evidence suggests that serotype C may be extinct in nature [2]. FMDV exhibits antigenic plasticity, resulting in multiple sub-types for each serotype, and this must be accommodated for when selecting an appropriate vaccine for use in the field [3]. The FMDV capsid comprises four structural proteins (VP1–4), three of which are externally facing and subject to the binding of antibodies which develop upon exposure to the intact virus antigen. In addition to the neutralising antibodies which develop against the characterised antigenic sites, non-neutralising antibodies targeting external and internal epitopes are also generated after vaccination and/or infection [4,5,6]. 

Determining the correlates of protection against viral diseases is crucial in order to assess the impact of vaccines [3]. In common with many other viruses, FMDV protection correlates with the titre of antibodies as measured using a virus neutralisation test (VNT). In general, high VNT titres against a specific virus will provide good protection against a homologous strain [7,8]. However, the VNT specifically measures neutralising antibodies rather than the entire population. Increasingly, there is a greater appreciation of the properties of an antibody beyond its ability to bind, with assays now measuring factors such as affinity as well as Fc-dependent functions [9,10]. 

Binding interactions of antibodies to their epitopes are measured in terms of affinity and avidity. Affinity is the term ascribed to the strength of a specific interaction between two molecules. In contrast, avidity refers to the overall binding among a collection of molecules. High-affinity binding is often identified as a hallmark of potently neutralising antibodies. Importantly, whereas affinity refers to the 1:1 interaction of an antibody paratope with its corresponding epitope, a polyclonal serum contains a plethora of different antibodies, each with a specific affinity. As a result, it is often better to assess the overall binding strength afforded by a collection of antibodies, i.e., avidity. Previous studies of avidity with reference to FMDV have suggested that cross-reacting antibodies demonstrate higher avidity [11], and that measuring avidity can enhance the precision of predicting cross-protection against heterologous strains [12].

Modified enzyme-linked immunosorbent assays (ELISAs) have frequently been used to measure antibody avidity, often resulting in an ‘avidity index’ (AI). Avidity ELISAs follow a similar format to conventional ELISAs but incorporate a wash step where either a single dilution or a series of dilutions of a reagent is used to remove weakly binding antibodies. However, whilst useful, avidity ELISA results based upon washes with chemicals to remove weakly binding antibodies can be confounded by the differing sensitivities of different epitopes; for example, antibodies targeting continuous epitopes can demonstrate greater resistance to the chemical (chaotrope) treatment relative to conformational epitopes [13].

Label-free alternatives to measuring avidity such as surface plasmon resonance (SPR) and bio-layer interferometry (BLI) allow the collection of kinetic data for both association and dissociation phases of antigen–antibody interactions in the absence of chemical agents. Explicit knowledge of interactant molarities allowing the affinity of interactions to be measured using SPR and BLI has resulted in these methods becoming a standard approach for the characterisation of monoclonal antibodies. Importantly, off-rates are a concentration-independent correlate of avidity. As a result, multiple studies have utilised both SPR and BLI in studies measuring antibody avidity against infectious agents as diverse as HIV-1, *Bacillus anthracis*, *Mycobacterium tuberculosis* and *Plasmodium falciparum* [14,15,16]. 

Here, we report the development of a novel, kinetics-based approach for the measurement of FMDV antibody avidity using BLI. We show that it is possible to utilise a peptide surrogate of a key antigenic loop on the surface of the FMDV capsid, as well as using entire capsids recombinantly expressed in vitro. Using antigen-loaded sensors, we demonstrate a relationship between the binding observed and neutralising titre, as well as an increase in avidity upon the challenge of vaccinated animals.

## 2. Materials and Methods

### 2.1. Antibody Samples

Previously characterised monoclonal antibodies (mAbs) targeting the FMDV capsid were obtained as supernatants of in vitro cultures of hybridomas grown at high density in small biofermenters (CELLine flasks). D9 is a neutralising mAb raised against the O1 Switzerland 1965 (Lausanne) strain of FMDV and targets the GH loop of type O viruses [17] (Table 1). D9 has a known escape mutation at VP1 position 144 within the GH loop (site 1). 2H6 is a neutralising antibody with specificity for the BC loop (site 2, Table 1). These mAbs were purified using protein A/G columns according to the manufacturer’s conditions (Thermofisher, Waltham, MA, USA), after which purified mAbs were quantified by absorbance using a NanoDrop (Thermofisher, Waltham, MA, USA). 

Polyclonal sera were obtained from the serum collection held by the WRLFMD at the Pirbright Institute. A selection of O1 Manisa positive sera were chosen according to the vaccination and/or challenge status of the animal, as well as their neutralising titres determined using a virus neutralisation assay as previously described [18].

### 2.2. ELISA

Ninety-six-well immunoassay plates (Maxisorb, Thermofisher, Waltham, MA, USA) were coated overnight at 4 °C with the appropriate ligand diluted in 50 µL of 0.05 M carbonate-bicarbonate coating buffer. For the detection of recombinant virus-like particles (VLPs), plates were coated with purified bovine αvβ6 recombinant integrin [19] diluted to 1 µg/mL. In order to bind biotinylated antigens, plates were coated overnight at 4 °C using 2.5 µg/mL streptavidin in coating buffer. The following day, plates were washed three times with phosphate-buffered saline (PBS) containing 0.05% Tween 20 (PBS-T). Non-specific binding was blocked for 1 h using blocking buffer (5% skimmed milk in PBS plus 1% equine serum). The blocked plates were incubated with VLPs in blocking buffer for 1 h at room temperature prior to washing in PBS-T.

Primary antibodies were incubated at the indicated dilutions for 1 h in blocking buffer, washed in PBS-T and incubated with a horse radish peroxidase (HRP)-conjugated anti-species secondary antibody (Invitrogen, Waltham, MA, USA) for a further 1 h. Following a final wash with PBS-T, plates were developed for 15 min in BioFX^®^ TPB (Cambridge Bioscience, Cambridge, UK) and stopped using 0.6 M sulphuric acid. Absorbance was read at 450 nm.

### 2.3. Recombinant Empty Capsids and Biotinylated Peptide

A biotinylated peptide representing antigenic site 1 was used as a surrogate target to mimic specific antibody interactions with the viral capsid. Here, we used a biotinylated peptide representing amino acids 134–157 of the O1 Kaufbeuren VP1 protein encompassing the GH loop and Arg-Gly-Asp (RGD) motif responsible for interacting with the integrin receptor on target host cells (Table 1). Biotinylated peptides were synthesised commercially (Peptide Protein Research Ltd., Fareham, UK) as previously described [20].

VLPs, also referred to as 75S particles, are generated by the expression of the viral structural proteins alongside the viral 3C protease. The viral proteins self-assemble into complete capsids which can then be purified via ultracentrifugation. 

HEK293T cells (kindly provided by Dr Claudine Porta, University of Oxford, ATCC, CRL-3216) were maintained in Dulbecco’s modified Eagle’s medium (DMEM, Gibco) supplemented with 10% foetal bovine serum and 5% penicillin/streptomycin (Gibco). VLPs were produced using a vaccinia virus (VV) system as previously described [21]. Briefly, HEK293T cells were grown to confluence in roller bottles prior to infection with a recombinant VV expressing the FMDV P1 region alongside the FMDV 3C protease, under the control of the T7 promoter, and a recombinant VV expressing the T7 polymerase. After 24 h incubation, cells were pelleted, resuspended and lysed by the addition of 0.5% Igepal. Capsids were concentrated through a 30% sucrose cushion and then further purified by centrifugation through a 15–45% sucrose gradient. Fractions containing capsids were combined and concurrently concentrated, and the buffer was exchanged into PBS using Amicon centrifugal filter units (Merck, Darmstadt, Germany) according to the manufacturer’s instructions. Capsids were biotinylated using the lightning-link type B kit (AbCam, Cambridge, UK) according to the manufacturer’s instructions using 20 µg capsid per reaction. Following the addition of the neutralisation reagent, biotinylation reactions were subjected to buffer exchange into HBS-EP buffer (10 mM Hepes, 150 mM sodium chloride, 3 mM EDTA, 0.005% Tween 20, pH 7.4) (Teknova, Hollister, CA, USA) using Zebaspin 40K columns. The capsid concentration was determined using the Qubit protein assay kit.

### 2.4. The Impact of Biotinylation upon Antigenicity

To assess the impact of biotinylation upon antigenicity, ELISA plates were first coated with recombinant soluble αvβ6 integrin [19] to facilitate the presentation of FMDV particles. Following the binding of VLPs to the integrin, the FMDV-specific mAb D9 was used as a primary antibody followed by detection using an HRP-labelled secondary antibody. 

### 2.5. Bio-Layer Interferometry

BLI experiments were performed using the Octet R8 8-channel instrument with streptavidin (SA) biosensors (Sartorius). All incubations were performed at 30 °C with 1000 rpm shaking. Biosensors were functionalised with optimal levels of FMDV antigens (referred to as ligands in BLI, Table 1) by performing scouting experiments. Dilutions of the biotinylated capsid in HBS-EP buffer were each assessed for their ability to (a) load onto the surface of the biosensor and (b) produce a sufficient signal (binding) upon dipping into the antibody. The immobilisation incubations were as follows: (1) 60 s at baseline (HBS-EP); (2) 900 s in the diluted ligand; (3) a 120 s wash step in HBS-EP. Functionalised sensors were either used immediately or stored in HBS-EP at 4 °C. A 10 min incubation with a shaking step was included at the start of all runs to ensure all reagents had reached 30 °C prior to experiments commencing.

Dilution series of mAbs or sera (Table 1) were prepared in HBS-EP in 96-well back plates (Greiner Bio-One, Stonehouse, UK). Kinetics experiments were performed using the following incubations: (1) 60 s in HBS-EP (baseline); (2) 1200 s in diluted antibody/serum (association); (3) 1200 s in the same well as used for the baseline (dissociation). Every run included a reference well (loaded sensor with all incubations being blank buffer). Double referencing to account for non-specific interactions with the biosensor was performed by generating association/dissociation data for unloaded sensors as well as those functionalised with ligands. 

All data were analysed using the Octet Analysis Studio 12.2 software. The response levels observed in BLI correspond to the level of binding, and a minimum response value of 0.1 nm is required to reliably model BLI curves; thus, samples demonstrating binding of <0.1 nm were removed from the analysis step. Data were modelled using the 1:1 global model within the Octet software. Full R^2^ values of >0.98 were accepted as high-quality data indicative of a reliable result. BLI measurements were obtained for three dilutions of serum (1:80, 1:160 and 1:320) using sensors loaded with O1 Manisa VLPs.

### 2.6. Octet Biosensor Loading

**Figure 1:**
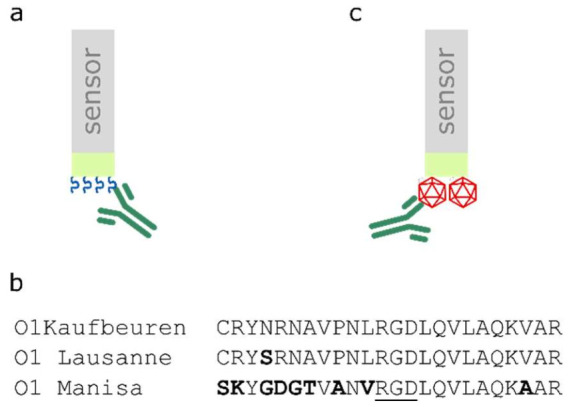
(**a**) Biotinylated peptides (blue) are loaded onto the streptavidin-coated Octet biosensor tip (lime) prior to dipping into antibody samples (green). (**b**) Amino acid sequences of GH-loop peptides relevant to this study. (**c**) Cartoon depicting the strategy of loading biotinylated O1 Manisa recombinant FMDV capsids (red) onto Octet biosensors.

An initial ‘scouting’ experiment was performed to optimise the loading of Octet sensors with the biotinylated peptide (Figure 1a,b). A two-fold dilution series was performed using peptide concentrations ranging from 4.69 to 300 nM. Sensors were loaded for 900 s before testing reactivity with the purified D9 mAb. 

Scouting experiments were also performed to determine the optimal concentration of the biotinylated O1 Manisa FMDV capsids (Figure 1c) with which to load the biosensors, using a dilution series that ranged between 20 µg/mL and 2.5 µg/mL. Antibody interactions with the functionalised biosensors were assessed using FMDV-specific sera raised against O1 Manisa diluted 1:150 in assay buffer.

### 2.7. Non-Specific Binding

To assess the impact of non-specific binding (NSB) on the BLI measurements, we evaluated the response levels obtained using unloaded sensors. Sensors were either loaded with the capsid or incubated in assay buffer for the equivalent time prior to being dipped into equal dilutions of O1 Manisa positive polyclonal sera derived from three different animals. In an attempt to remove NSB (and thus avoid the necessity for reference sensors), we titrated Tween 20 into the assay buffer.

### 2.8. Statistics

All statistical analyses were performed using R [22]. The Wilcoxon rank-sum test was used to compare different classes using vaccinated and vaccinated/challenged as different treatments. Correlative relationships between parameters were tested using Pearson’s correlation test. 

## 3. Results

### 3.1. Biosensor Loading with Surrogates of FMDV Antigens

**Figure 2:**
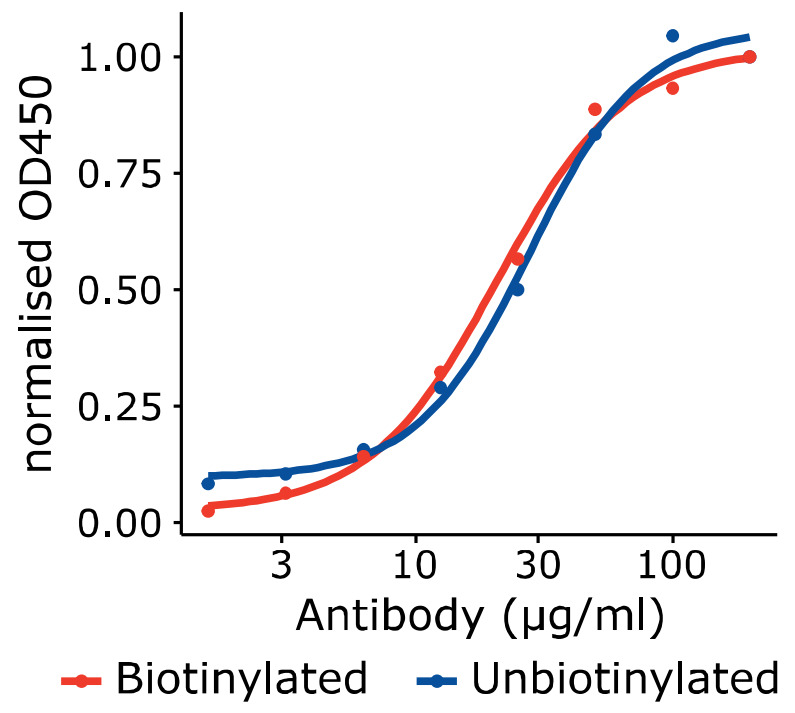
The impact of biotinylation upon the antigenicity of recombinant capsids.

First, we assessed the possibility that biotinylation could impact the antigenicity of the VLPs. Using an ELISA approach, we observed no significant differences between the curves obtained using either biotinylated or non-biotinylated capsids, providing evidence that the biotinylation procedure did not negatively impact upon the antigenic characteristics of the VLPs (Figure 2).

**Figure 3:**
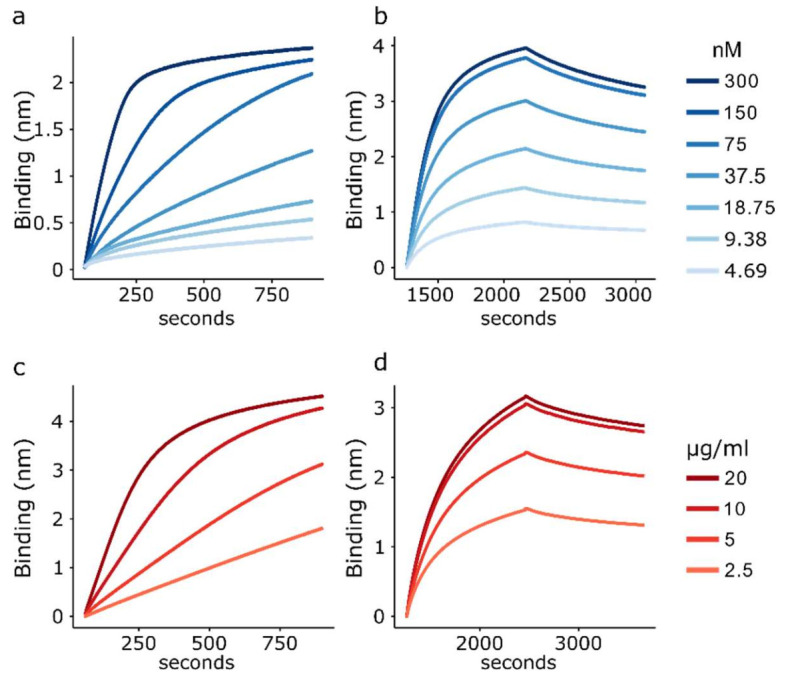
Biosensor loading with FMDV antigens. (**a**) BLI curves reflecting the loading of biotinylated peptide (ligand) onto the sensor surface. (**b**) Association and dissociation curves reflecting the interactions between Octet biosensors loaded with peptide and the D9 monoclonal antibody. Note: the 150 and 75 nM curves overlap. (**c**) Response levels observed when loading biosensors with different concentrations of recombinant capsid (2.5–20 µg/mL). (**d**) Reactivity of O1 Manisa polyclonal sera with biosensors functionalised with the different amounts of recombinant capsid.

Scouting experiments were performed to determine the optimum concentration of biotinylated ligands to use for loading Octet biosensors. GH-loop peptide concentrations of 300 and 150 nM resulted in complete saturation of the sensor surface (as indicated by plateauing in the loading phase), thus resulting in non-ideal conditions for performing kinetics experiments. The lower peptide concentrations indicated that saturation occurred at between 75 and 150 nM (Figure 3a). An increase in the response was observed when using the D9 monoclonal antibody and a peptide concentration of 9.375 nM (Figure 3b); thus, a concentration of 10 nM was used in further experiments as a compromise between a sufficient response (indicative of binding) and the risk of both arms of the antibody binding peptides. 

Sensor loading was observed for all of the O1 Manisa capsid concentrations trialed, and binding was observed using polyclonal serum (Figure 3c,d). A concentration of 5 µg/mL was taken forward as the optimal concentration, providing a compromise between a sufficient response and efficient use of the recombinant capsid.

### 3.2. Non-Specific Binding

**Figure 4:**
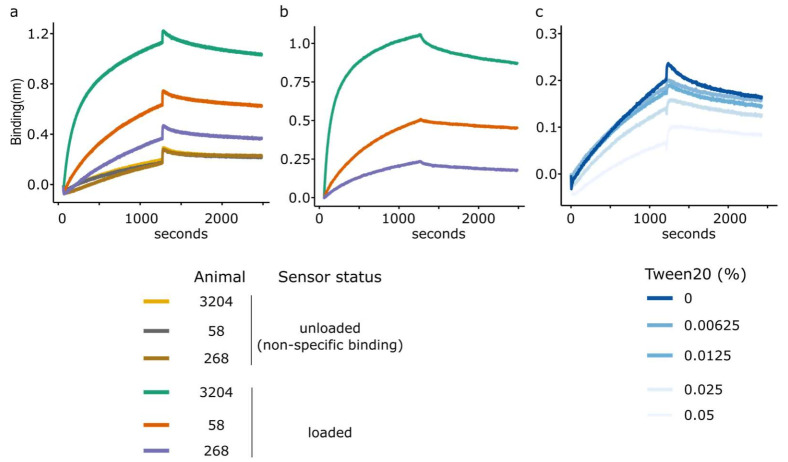
Non-specific binding (NSB) to Octet biosensors. (**a**) Un-referenced BLI association/dissociation curves indicating the difference between the loaded and unloaded sensors (dark colours) highlighting NSB for three bovine sera (3204, 58 and 268). (**b**) Double referenced curves following the subtraction of the unloaded reference sensors. (**c**) Non-specific interactions in serum from sample 3204 (diluted 1:100) with unloaded sensors in kinetics buffer modified with various concentrations of Tween 20 giving a range of 0.00625–0.05%.

Accurate BLI kinetics experiments rely upon carefully measuring the activity associated with a specific concentration of sera with functionalised biosensors, with an assumption that 100% of the observed response is derived from the interaction between the ligand and analyte. However, the complex nature of polyclonal antibody binding means that background interactions can interfere with the precision of measurements. Whilst significant binding was observed using capsid-loaded sensors, some binding was also evident for each of the unloaded sensors, indicative of non-specific binding (Figure 4a). Subtraction of this background binding signal obtained using unloaded (reference) sensors from the signal observed with loaded sensors leads to normalised traces which can be used for kinetics experiments (Figure 4b). 

The addition of Tween 20 removed some, although not all, of the NSB (Figure 4c). Increasing NaCl concentration also failed to adequately remove the NSB (data not shown). Given these difficulties, it was decided that subsequent kinetics experiments would be performed using unmodified buffers in order to maintain consistency, but to use reference sensors and double referencing for subsequent kinetics experiments.

### 3.3. Site 1 Specific Interactions Using the GH Loop

Using biosensors functionalised with the optimal concentration of GH-loop peptides, we tested the specificity of the GH loop as a surrogate for antigenic site 1 by comparing the binding of mAbs D9 and 2H6 that target sites 1 and 2, respectively. Antibody reactivity for both D9 and 2H6 against type O capsids was first confirmed by ELISA using recombinant VLPs (captured using recombinant integrin) or the GH loop (captured using streptavidin) as a ligand. 

**Figure 5:**
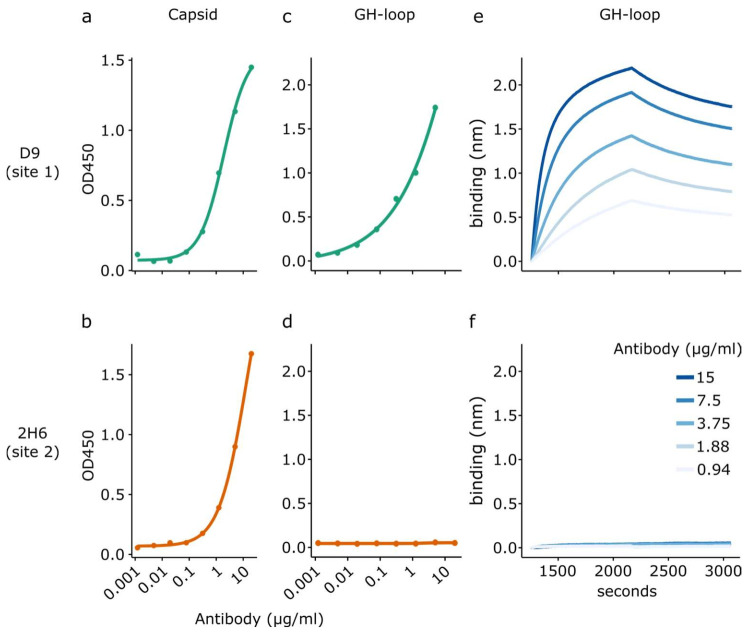
Measurement of specific interactions using a peptide surrogate of the GH loop. ELISA reactivity of the monoclonal antibodies D9 (**a**) and 2H6 (**b**) targeting antigenic sites 1 (GH loop) and 2, respectively, with complete recombinant capsids. ELISA reactivity of the monoclonal antibodies D9 (**c**) and 2H6 (**d**) targeting antigenic sites 1 (GH loop) and 2, respectively, with GH-loop peptides. BLI association and dissociation data for antibodies D9 and 2H6 when using the GH loop as a ligand (**e**,**f**).

As expected, both mAbs demonstrated reactivity against the entire FMDV capsid (Figure 5a,b). 

In contrast, only D9 showed binding to the GH loop in ELISA, with no indication of binding of 2H6 to the GH loop (Figure 5c,d). When used in BLI experiments using the GH loop as a ligand, the pattern of interactions mirrored the ELISA, with reactivity with the GH loop only being observed with D9 (Figure 5e,f). 

### 3.4. Evolution of Antibody Avidity during Vaccination/Challenge

The off-rate (Kdiss) measured during the dissociation phase of the BLI assay is a direct correlate of avidity and, importantly, is independent of the concentration. To assess the utility of BLI for use in FMDV serology studies, we tested a selection of cattle sera from a bank of characterised O1 Manisa vaccinal sera available at the Pirbright Institute. Sera were selected to cover a range of neutralisation titres and included three cattle which had only been vaccinated alongside seven animals which had been challenged with the homologous virus following vaccination. 

**Figure 6:**
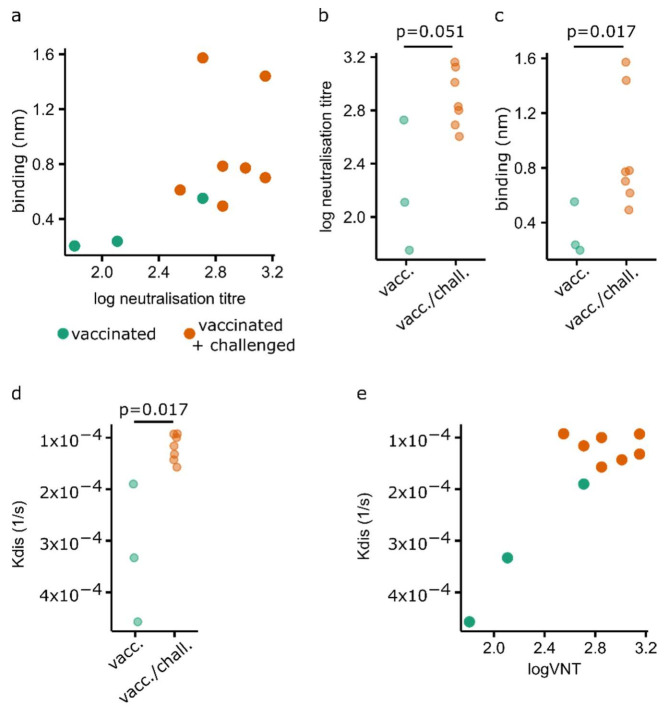
Binding and avidity characteristics of FMDV seropositive bovine serum. (**a**) The response values (nm) observed in BLI for different sera plotted against virus neutralisation titre (VNT). (**b**) Neutralisation titres of sera derived from vaccinated and vaccinated/challenged animals. (**c**) BLI binding values for sera derived from vaccinated and vaccinated/challenged animals. (**d**) Dissociation rates for sera derived from vaccinated and vaccinated/challenged animals. (**e**) Correlation between neutralisation titre and BLI dissociation rates.

For two out of the three vaccinated-only animals, less than three measurements were obtained which met the defined criteria and were thus not included in the calculations. The levels of binding (calculated as the median level of response) were plotted relative to the virus neutralisation titre (Figure 6a). 

Using this approach, a trend existed whereby sera with higher VNT titres demonstrated greater levels of binding (Figure 6a). However, the relationship between the median response and log VNT titre did not reach statistical significance (*p* = 0.063), reflecting a high level of variability among the different sera. A general trend was observed in which it was more difficult to detect capsid binding antibodies in sera from animals which had been vaccinated only (with no further challenge). In the panel of sera tested in this study, vaccinated-only animals had an overall lower neutralising titre than animals which had been subsequently challenged with the homologous strain (Figure 6b, *p* = 0.051). This was again reflected in the level of binding observed with BLI, which was significantly higher among vaccinated and challenged animals as compared to vaccinated-only animals (Figure 6c, *p* = 0.017). In addition to the greater binding observed with challenged vaccinees, the Kdiss values obtained using BLI indicated significantly greater levels of avidity (lower Kdiss values) in animals which had been challenged after vaccination (Figure 6d, *p* = 0.017). Interestingly, when Kdiss values were assessed relative to the VNT titre, a significant positive correlation was observed between VNT and avidity (*p* < 0.001). A clear trend existed whereby challenged animals all demonstrated a high level of avidity (range 9.26 × 10^−5^–1.57 × 10^−4^), in contrast to a wide range in avidity observed for animals which had only been vaccinated (range 1.9 × 10^−4^–4.5 × 10^−4^, Figure 6d,e).

## 4. Discussion

Infection by FMDV is initiated by viral particles binding to the αv integrin receptors (most prominently αvβ6) on host cells via a conserved Arg-Gly-Asp (RGD) motif located on the βG–βH loop present in the viral VP1 capsid protein [23]. The GH loop is commonly recognised as one of the key immunodominant sites on the FMDV particle and often referred to as site 1 [24]. At least a further four sites have been characterised using monoclonal antibodies [4,6,17], with ongoing research into novel epitopes on the virus capsid revealing further sites within VP2 and VP3 [20,25,26]. 

Previous attempts have been made to generate peptide-based vaccines. However, whilst these vaccines have shown promise, complete protection is not always achieved in the natural host [27,28,29,30,31,32,33,34]. In contrast, the currently available FMDV vaccines are based upon inactivated, complete virus particles. Humoral responses are the key arm of immunity that governs protection against FMDV, and understanding the specific components of sera is of fundamental importance. It is thus important to be able to interrogate the avidity of a polyclonal response against the entire anti-FMDV surface repertoire of epitopes.

The strength (affinity) of antibody binding to its epitope can directly impact the efficiency with which it blocks infection. Modern approaches to sequencing antibody repertoires, as well as high-throughput methods by which to express and characterise individual antigen-specific antibodies, are beginning to uncover the contribution of individual antibodies [35,36]. However, by definition, polyclonal serum represents a diverse collection of different antibodies, each with a specific affinity. It is increasingly acknowledged that there is variability in the efficacy of individual antibodies targeting specific epitopes as well as a greater awareness of the multifaceted nature of these responses [9,10,37]. The collective binding of different antibodies, referred to as avidity, is thus an important metric to characterise with regard to polyclonal sera. 

In this study, we utilised two forms of antigen/ligand: (i) a peptide representing a linear epitope present on the FMDV capsid surface and (ii) biotinylated recombinant FMDV capsids. The ability to measure and dissect antibody responses against specific sites expressed on the capsid may help understand the relationship between antibody titres and protection. 

The results presented here demonstrate that a simple peptide can substitute for site 1 in BLI experiments and allows the binding strength of both monoclonal and polyclonal sera to be captured. Interestingly, the GH loop sequence used in these experiments was that of O1 Kaufbeuren and contains multiple mismatches from that of O1 Manisa, the vaccine against which the polyclonal sera used in this study were raised. It is conceivable that the observed difference in avidity when using the O1 Kaufbeuren peptide rather than complete FMDV capsids (O1 Manisa VLP) is a result of the heterotypic nature of the ligand (O1K) and analyte (O1M) (62.5% amino acid identity). Further work is required to more closely define the relative contributions of the heterogeneity between the peptide ligand and analyte, versus the affinity values of antigen-specific antibodies. The O1 Kaufbeuren peptide was used due to its availability and use as an antigen in other laboratory procedures, and an O1 Manisa peptide will be required to fully interrogate the impact of mismatches. 

Humoral responses to FMDV result in both neutralising (as measured by VNT) and non-neutralising antibodies targeting the FMDV capsid, with neutralising titres showing the strongest correlation with protection [3]. In turn, previous observations have shown that VNT titres correlate strongly with the titre of total capsid-binding antibodies [3]. In this study, we observed a positive correlation between neutralising titres and antibody avidity as measured using BLI. In contrast, the level of binding observed in the BLI experiments did not correlate as strongly with VNT. These findings may suggest that the proportion of total antibodies that are neutralising versus non-neutralising may vary between sera. Interestingly, previous label-free studies have also reported a lack of a direct relationship between off-rates and ELISA [14]. Defining the exact protective efficacy of a serum is further complicated by the potential for non-neutralising antibodies to offer a level of protection via Fc-mediated functions: for example, opsonisation [38]. Assessing the kinetics of more extensive panels of capsid-specific antibodies, alongside structural studies, will shed further light upon the rules governing antibody-based protection against FMDV. 

Importantly, the capsids used in these experiments were covalently stabilised [21]. A further benefit of using recombinantly produced VLPs is that their production abrogates the requirement for high-containment facilities. It is possible that an inactivated, purified virus could be biotinylated and utilised in an analogous fashion to the VLPs used here. Indeed, it has previously been demonstrated that biotinylated virus particles can be used in a variety of assays [39]. However, the FMDV particle is inherently unstable and is easily disrupted by heat or alterations in pH. In this study, we performed kinetics experiments at 30 °C, a temperature at which native FMDV particles may begin to disassemble after an extended period of time, exposing internal epitopes. Thus, the use of covalently linked capsids ensures that the repertoire of epitopes was limited to those externally exposed on the 75S particle. We observed that 5 µg/mL capsid was a suitable loading concentration, both giving a sufficient signal (indicative of binding) for the majority of samples and avoiding oversaturation of the sensor surface (which can reduce the accuracy of binding measurements given the bivalent nature of antibodies). On the other hand, the necessity to achieve consistent levels of loading suggests that scouting experiments should be performed with different batches of ligands to ensure that results remain consistent. In addition, the impact of temperature on kinetics experiments should also be assessed.

The panel of sera used in this study comprised samples collected from vaccinated animals as well as those collected from vaccinated cattle followed by challenge using the homologous virus. As expected, an increase in the antibody neutralising titre was observed following challenge of the vaccinated animals. The change in titre was also captured using BLI, with a significantly increased level of binding observed, suggesting that a threshold BLI response may be sufficient to reflect the VNT titre and, in turn, the level of protection. On the other hand, the avidity also appears to increase following challenge. This reflects a previous observation by Lavoria et al., who observed an increase in the ELISA avidity index following vaccination and boost with FMDV serotypes type A, O and C [11]. 

It has previously been suggested that the isotype IgG1 is of particular significance in the protection afforded by vaccines against FMDV [11,40]. It is possible that the BLI assay described here can be extended to define the relative contribution of different antibody isotypes in order to further dissect this concept, and previous studies have demonstrated the option to include a third stage using isotype-specific antibodies [41,42].

It is important to note that the results presented here are based upon a limited number of vaccinated animals, and more extensive testing, including analysing sera derived from infected animals, is required in order to fully disentangle the relative contributions of the titre and avidity to protection. The study described here deliberately focused upon O1 Manisa, one of the canonical FMDV vaccine strains. However, it will be interesting to observe and measure heterogeneity using BLI when using non-homologous sera against a specific antigen. In turn, these measurements may enhance the precision of current vaccine matching protocols.

In summary, we have demonstrated that it is possible to use BLI to measure the avidity of bovine antibodies against the FMD viral capsid. BLI represents a useful approach alongside chaotrope-based ELISAs for the characterisation of antibody responses to FMDV.

## Figures and Tables

**Table 1:** Reagents and their role during bio-layer interferometry experiments.

Reagent	Description	Antigenic Site Targeted	Function in BLI
O1 Manisa VLPs ^1^	Empty capsids	N/A ^2^	Ligand
GH-loop peptide	Biotinylated peptide	N/A	Ligand
D9	Monoclonal antibody	Site 1	Analyte
2H6	Monoclonal antibody	Site 2	Analyte
O1 Manisa serum	Polyclonal serum	Multiple	Analyte

^1^ VLPs were biotinylated prior to use in BLI studies. ^2^ Not applicable

## Data Availability

Not applicable.
